# Editorial: The gut-liver axis in MASLD: microbiome, metabolism, disease progression and therapeutic pathways

**DOI:** 10.3389/fmed.2026.1947178

**Published:** 2026-10-01

**Authors:** Zhengyang Bao

**Affiliations:** Wuxi Maternity and Child Health Care Hospital, Wuxi, China

**Keywords:** gut-liver axis, MASLD, metabolic dysfunction, microbiome, precision medicine

Metabolic dysfunction-associated steatotic liver disease (MASLD) is now recognized as the most prevalent chronic liver disease worldwide and represents a rapidly growing public health burden. The 2023 multisociety nomenclature replaced non-alcoholic fatty liver disease (NAFLD) with MASLD to establish a positive diagnosis based on hepatic steatosis and cardiometabolic risk rather than an exclusion-centered definition (1). Recent meta-analytic data using the steatotic liver disease framework estimate a global MASLD prevalence of approximately one-third of adults, although substantial heterogeneity exists across regions, populations, and diagnostic methods (2, 3). MASLD spans hepatic steatosis without steatohepatitis, metabolic dysfunction-associated steatohepatitis (MASH), fibrosis, cirrhosis, and hepatocellular carcinoma (HCC), and is closely intertwined with obesity, type 2 diabetes, dyslipidemia, and cardiovascular disease (2, 4).

Within this broader metabolic context, the gut-liver axis offers a biologically plausible framework linking intestinal ecology to hepatic injury. Gut-derived microbial products and metabolites, bile acids, nutrients, and inflammatory signals reach the liver through the portal circulation, while hepatic bile-acid metabolism and systemic metabolic status reciprocally shape the intestinal environment (5). Experimental studies support mechanisms involving intestinal barrier dysfunction, endotoxin exposure, altered short-chain fatty acid and bile-acid signaling, immune activation, and metabolic stress. In humans, however, dysbiosis may be both a contributor to and a consequence of diet, obesity, diabetes, medication exposure, and liver dysfunction. Accordingly, association, mechanistic plausibility, and demonstrated causality should be distinguished throughout the interpretation of microbiome findings in MASLD (2, 5).

This Research Topic was designed to examine gut-liver communication across microbial ecology, host metabolism, immune regulation, disease progression, biomarker discovery, and intervention. The ten contributions span narrative reviews, animal mechanistic studies, human clinical investigations, a case report, and a machine-learning cohort study (Dua et al.; Dongkun et al.; Huang et al.; Wang et al.; Ran et al.; Chen et al.; Geng et al.; Cui et al.; Song et al.; Guan et al.). These study types do not provide equivalent levels of inference. We therefore interpret the Research Topic thematically and according to evidence strength, rather than as a sequence of parallel findings.

At the mechanistic level, the contributions collectively support a network model in which intestinal barrier integrity, microbial metabolites, bile-acid signaling, insulin resistance, and immune responses interact with hepatic lipid metabolism. Dua et al. synthesized evidence connecting gut microbial dysbiosis with insulin resistance, type 2 diabetes, intestinal permeability, and systemic inflammation, providing a metabolic-microbiome framework for MASLD. Dongkun et al. added preclinical evidence from a rat model, in which different aerobic exercise loads were accompanied by changes in lipid metabolism, intestinal barrier function, bile-acid-related signaling, and butyrate-producing taxa; medium- and high-load exercise performed better than low-load exercise for several metabolic outcomes. These findings support further investigation of exercise dose and gut-liver mechanisms, but they should not be interpreted as a clinical exercise-prescription recommendation. Ran et al. reviewed the IL-33/ST2 axis as a stage- and context-dependent link among metabolic stress, intestinal barrier function, immune regulation, fibrosis, and tumor development. Importantly, the potentially protective and pathogenic actions of IL-33 underscore why bidirectional immunobiology may complicate therapeutic targeting. Chen et al. introduced a temporal dimension through the circadian rhythm-gut microbiota-liver axis, proposing that feeding time, host clocks, microbial rhythmicity, and metabolite production may jointly influence MASLD biology; however, time-restricted feeding, chronobiotic probiotics, postbiotics, and circadian-timed pharmacotherapy remain areas for prospective clinical validation rather than established MASLD treatments.

The collection also illustrates both the appeal and the current limitations of microbiome-related biomarkers. Huang et al. reported clinical improvement in hepatic fat deposition together with changes in tongue-coating microbial profiles after washed microbiota transplantation (WMT), including decreases in Peptostreptococcus and increases in potentially beneficial taxa such as Lachnospiraceae and Bifidobacterium. The study used the MAFLD terminology, meaning metabolic dysfunction-associated fatty liver disease, which overlaps substantially with but is not identical to current MASLD criteria. Moreover, tongue-coating communities are oral microbial readouts rather than direct measurements of the intestinal microbiome, and the study design does not support strong causal attribution between the observed microbial changes and hepatic improvement. Outside this collection, prospective metagenomic-metabolomic studies have shown that microbiome features can contribute to future fatty-liver risk prediction and that MASLD is associated with transkingdom microbial, transcriptomic, and metabolomic changes (6, 7). These findings support continued development of microbiome-informed biomarkers, but individual taxa or taxonomic signatures are not yet sufficiently validated for routine MASLD diagnosis or risk stratification. Functional pathways and reproducible metabolite profiles may ultimately prove more transferable across cohorts than single bacterial taxa.

Intervention studies in this Research Topic cover lifestyle modification, microbiota transplantation, natural products, and chronobiology, but their translational maturity differs considerably. Dongkun et al. provide preclinical evidence that exercise-associated metabolic benefits may involve the gut-liver axis, whereas the WMT study by Huang et al. offers human interventional observations that require confirmation in randomized studies with standardized quantitative liver endpoints and longer follow-up. Wang et al. reviewed Schisandra chinensis Fructus and its active metabolites, describing potential effects on hepatic lipid accumulation, insulin sensitivity, oxidative stress, and related pathways; the supporting evidence remains predominantly preclinical and mechanistic, so these compounds are better regarded as therapeutic candidates than established clinical therapy. Similarly, the chronobiological approaches discussed by Chen et al. are mechanistically attractive but clinically immature. Importantly, a randomized trial of allogenic fecal microbiota transplantation in NAFLD did not significantly improve HOMA-IR or MRI-measured hepatic fat, although intestinal permeability improved in a small subgroup with abnormal baseline permeability (8). More recent work on resistant starch also demonstrates substantial interindividual variability in microbiome-mediated treatment response, supporting phenotype- and microbiome-based patient stratification rather than a one-size-fits-all strategy (9).

Microbiota transplantation additionally raises safety and implementation issues that are especially important when the target disease already has established lifestyle and cardiometabolic management pathways. Donor screening, pathogen and antimicrobial-resistance transmission, preparation and delivery standardization, regulatory oversight, and the durability of engraftment all require careful consideration. Donor-derived extended-spectrum beta-lactamase-producing Escherichia coli bacteremia has previously been genomically linked to fecal microbiota transplantation, including a fatal case, underscoring the need for stringent safety procedures (10). At present, WMT/FMT should therefore be viewed as an investigational strategy in MASLD rather than routine care.

Disease progression and extrahepatic consequences broaden the scope of the collection but also require clear boundaries of interpretation. Geng et al. reviewed metabolic dysregulation in MASLD-associated HCC, integrating glucose and lipid metabolism, oxidative stress, chronic inflammation, bile acids, short-chain fatty acids, and oncogenic signaling networks. This contribution supports the concept that gut-liver research should extend longitudinally from early metabolic dysfunction to fibrosis and carcinogenesis. By contrast, Cui et al. described a mixed-etiology cholestatic liver-injury case in the setting of heavy alcohol exposure, metabolic dysfunction, and suspected secondary iron overload. Its relevance to the Topic lies primarily in illustrating real-world etiologic overlap and diagnostic heterogeneity, not in demonstrating a microbiome-mediated mechanism of MASLD. Song et al. developed a machine-learning model for overt hepatic encephalopathy after transjugular intrahepatic portosystemic shunt in cirrhosis. Because the model did not center on MASLD etiology or microbiome variables, its connection is best viewed as a methodological extension toward individualized risk stratification in advanced liver disease rather than direct evidence for the gut-liver axis. Finally, Guan et al. showed in high-cholesterol-fed LDLR-/- mice that temporal gut microbiota dysbiosis was associated with impaired microbial metabolism, systemic inflammation, LDL desialylation, and accelerated atherosclerosis. This work supports the broader cross-organ concept that microbiome-metabolism-immune networks may contribute to cardiometabolic complications, but findings from an experimental atherosclerosis model should not be directly extrapolated to MASLD patients.

Taken together, the principal contribution of this Research Topic is not the identification of a single reproducible “MASLD microbiome,” but the clarification of a biologically credible yet highly heterogeneous gut-liver communication framework. Experimental models provide manipulable evidence for specific mechanisms; human observational studies primarily establish association and prediction; and human interventional evidence remains comparatively limited. This evidence hierarchy argues for a microbiome-informed, rather than microbiome-centered, approach to precision medicine. Microbial features may add value when integrated with metabolic phenotype, imaging, fibrosis assessment, host factors, and metabolite profiling, but they do not currently replace validated MASLD risk-stratification and management pathways, which remain centered on fibrosis assessment, lifestyle intervention, and treatment of cardiometabolic comorbidities (2).

Several barriers continue to limit clinical translation. Gut microbiome composition and function vary with geography and ethnicity, diet and alcohol exposure, age, obesity and diabetes severity, and medication use. Antibiotics are obvious modifiers, but non-antibiotic drugs such as metformin and proton-pump inhibitors are also associated with substantial microbiome differences; incretin-based therapies may further modify weight, diet, gastrointestinal physiology, and metabolic context and should be carefully captured in future studies (11). Technical variation adds another layer: stool collection timing, storage, DNA extraction, 16S rRNA vs. shotgun metagenomic sequencing, bioinformatic pipelines, compositional data structure, and batch effects can all alter reported signatures. The STORMS checklist provides a useful framework for improving transparency and reproducibility in human microbiome studies (12). These challenges help explain why taxonomic signatures often fail to reproduce across cohorts and reinforce the need to prioritize externally validated microbial functions, metabolites, and host-microbe composite features.

Future progress will require several coordinated steps. Multicenter longitudinal MASLD cohorts should standardize microbiome and metabolome collection while prospectively recording diet, alcohol, medications, metabolic phenotype, fibrosis status, and clinically meaningful outcomes. Candidate mechanisms should be triangulated across prospective human associations, gnotobiotic or defined-community models, microbiota-transfer experiments, and direct perturbation of candidate metabolites or host pathways. Microbiome-targeted trials should be phenotype-stratified, appropriately controlled, and designed to evaluate durability, safety, liver fat, fibrosis, and patient-relevant outcomes. Finally, multimodal prediction models integrating clinical variables, imaging, host metabolomics, and microbial functional features require independent external validation and demonstration of incremental clinical utility before incorporation into decision pathways.

In conclusion, this Research Topic positions gut-liver communication as a biologically plausible but heterogeneous biomarker and therapeutic framework for MASLD. Its clinical application will depend on stronger causal validation, methodological standardization, reproducibility across populations, and phenotype-specific prospective trials. The near-term opportunity is therefore not to replace current MASLD care with microbiome-based management, but to develop rigorously validated microbiome-informed tools that complement established metabolic, imaging, and fibrosis-based approaches. We extend our sincere gratitude to all authors, reviewers, and the Frontiers editorial team for their contributions, and we hope this collection stimulates research that moves the field from descriptive dysbiosis toward reproducible function, causal mechanisms, and safe, stratified clinical intervention.
